# Scapular dyskinesis is common among asymptomatic European basketball players at the professional level

**DOI:** 10.1002/ksa.70060

**Published:** 2025-09-05

**Authors:** Alp Paksoy, Doruk Akgün, Jonas Pawelke, Larissa Eckl, Arda Mavi, Selda Uzun, Berhan Bayram, Murat Canbakal, Ugur Dilicikik, Murat Erdem, Nihat D. Demirkiran, Baris Kocaoglu

**Affiliations:** ^1^ Charité University Hospital, Center for Musculoskeletal Surgery Berlin Germany; ^2^ Justus Liebig University Clinic Gießen Germany; ^3^ Medical University Innsbruck Innsbruck Austria; ^4^ Acıbadem University School of Medicine Istanbul Turkey; ^5^ Faculty of Sport Sciences, Institute of Health Sciences Marmara University Istanbul Turkey; ^6^ Turkish Basketball Federation Istanbul Turkey; ^7^ Department of Orthopedics and Traumatology Kütahya Health Sciences University Kütahya Turkey

**Keywords:** asymptomatic professional basketball players, Kibler's classification, prevalence, professional overhead athletes, scapular dyskinesis

## Abstract

**Purpose:**

Scapular dyskinesis (SD) is present in as many as 67%–100% of athletes with shoulder injuries but it is also highly present in many asymptomatic individuals. The aim of the present study was to identify and analyse SD among asymptomatic professional basketball players.

**Methods:**

A total of 54 European professional basketball players of various professional levels and ages were included in this prospectively recruited cross‐sectional study. Participants were assessed using subjective shoulder value (SSV), visual analogue score (VAS) and active range of motion (ROM). Visual combined palpation was used to classify scapular position and movement patterns according to Kibler's method. The clinical examination was completed by evaluating potential coexisting instability (apprehension test, Kim/Jerk and O'Brien tests) and hyperlaxity (Beighton score).

**Results:**

The mean age of all participants (27 female, 27 male; 108 shoulders) was 23.9 ± 6.5 years. 28.7% of the included shoulders had SD (31/108; right: *n* = 12; left: *n *= 19), while none of the participants had a diagnosed SD before the present study. Shoulders with SD exhibited a significantly lower SSV (95.0 ± 10.5% vs. 99.0 ± 4.0%; *p* = 0.004) and reduced abduction (171.8 ± 11.7° vs. 176.6 ± 8.3°, *p* = 0.013) compared to shoulders without SD. Shoulders with at least one previous injury showed a significantly lower SSV compared to shoulders without previous injury (92.9 ± 12.0% vs. 98.4 ± 5.6%; *p* = 0.001). Shoulders with pain occurring at least once in last 12 months showed a significantly higher prevalence of SD (6/10 vs. 25/98; *p* = 0.022) and a lower SSV (90.5 ± 16.4% vs. 98.6 ± 4.4%; *p* = 0.024) compared to shoulders without pain in last 12 months.

**Conclusion:**

SD was observed in 28.7% of the shoulders in asymptomatic European professional basketball players. SD may represent a sport‐specific adaptation, but its association with reduced shoulder function and pain suggests clinical relevance, emphasising the need for early detection and intervention.

**Level of Evidence:**

Level III, cohort study.

**Abbreviations**
ABD: abduction
BMI: body mass index
C: center
ER: external rotation
FF: forward flexion
IR: internal rotation
MCID: minimal clinically important difference
PF: power forward
PG: point guard
ROM: range of motion
SAT: scapular assistance test
SD: scapular dyskinesis
SF: shooting forward
SG: shooting guard
SICK: scapular malposition, inferior medial border prominence, coracoid pain and malposition and dyskinesis of scapular movement
SRT: scapular retraction test
SSV: subjective shoulder value
VAS: visual analogue score

## INTRODUCTION

Scapular dyskinesis (SD) is defined as alterations in scapular positioning at rest as well as during dynamic movement including increased protraction with a prominent scapular medial border and an inferior angle, which may result in atypical and inefficient kinematics of the arm and shoulder [46, 51, 56]. Impaired scapulothoracic kinematics alters the glenohumeral alignment, overloads the compensatory musculature, limits shoulder strength and range of motion (ROM) and causes pain, leading to suboptimal shoulder function [14, 22, 41, 54, 55, 63, 64]. Alterations in scapular motion can occur due to fatigue, neurologic dysfunction, glenohumeral arthritis, injury of the acromioclavicular joint, rotator cuff pathology, clavicular fracture, subacromial impingement, glenohumeral instability, labral injury and adhesive capsulitis [1, 5, 8, 19, 27, 28, 47, 59, 61].

SD is present in as many as 67%–100% of athletes with shoulder injuries but it is also frequently observed in many asymptomatic athletes with a reported prevalence of approximately 42% [7, 29, 51]. It remains unclear if SD is a sports‐related pathology coexisting with shoulder dysfunctions due to repetitive trauma [22] or sports‐specific adoption that is potentially beneficial for maximal performance and protective against injury in athletes without symptoms [42]. The alterations caused by SD can increase the theoretical risk of injury and require a preventative intervention [4, 7, 10, 59]. Despite extensive research on SD in other overhead sports such as baseball, handball, rugby and swimming [4, 9, 23, 37, 39, 52], its prevalence in basketball players remain underexplored. This study aimed to assess the prevalence of SD in European professional basketball players and examine its potential impact on shoulder function. The hypothesis was that SD would be prevalent in asymptomatic overhead athletes and would be associated with reduced shoulder function.

## MATERIALS AND METHODS

### Study population

A prospectively recruited cohort of 54 professional basketball players (108 shoulders) who were playing at various European Basketball organisations including EuroLeague, EuroCup and FIBA National Basketball, without any actual symptoms of the upper extremities or thorax were included. The recruited athletes were assessed at rest, before any physical effort and at the end of the season, to obtain a realistic prevalence of SD excluding the influence of fatigue. Approval from the institutional ethics committee was obtained prior to onset of investigation (EA4/024/25).

### Clinical evaluation

In the clinical evaluation conducted by an experienced examiner, cohort characteristics were collected including body mass index (BMI), dominant hand, comorbidities and history of previous shoulder operations. Sports‐related information was documented, such as current professional level, playing position, years of professional experience and training frequency (h/day and h/week). Previous injuries were assessed, with a previous injury defined as any shoulder injury that led to missed training or required medical attention within the past 12 months.

After having been included in the study, both shoulders of each participant were assessed using patient‐reported subjective shoulder value (SSV) [13] and participants were asked about any recent shoulder pain or functional complaints using the following question: ‘Have you had, in the past 12 months, any pain and/or difficulty moving your shoulders that lasted more than one day?’. The pain levels of participants with positive responses at rest and exertional pain while performing were documented, utilising visual analogue score (VAS) [20]. Active ROM for both shoulders was recorded using a manual goniometer in a standardised manner for abduction (ABD), forward flexion (FF), external rotation (ER) and internal rotation (IR). ER was measured with the patient standing and the arm positioned at the side, while IR at 0° ABD was determined based on the thumb's reach to different levels of the spine and illustrated in points according to the vertebral level (L3: 2, L2: 4, L1: 6, T12: 8; T11: 10, T10: 12, T9: 14, T8: 16, T7: 18, T6: 20). Additionally, both ER and IR were evaluated with the shoulder in 90° abduction.

**Figure 1:**
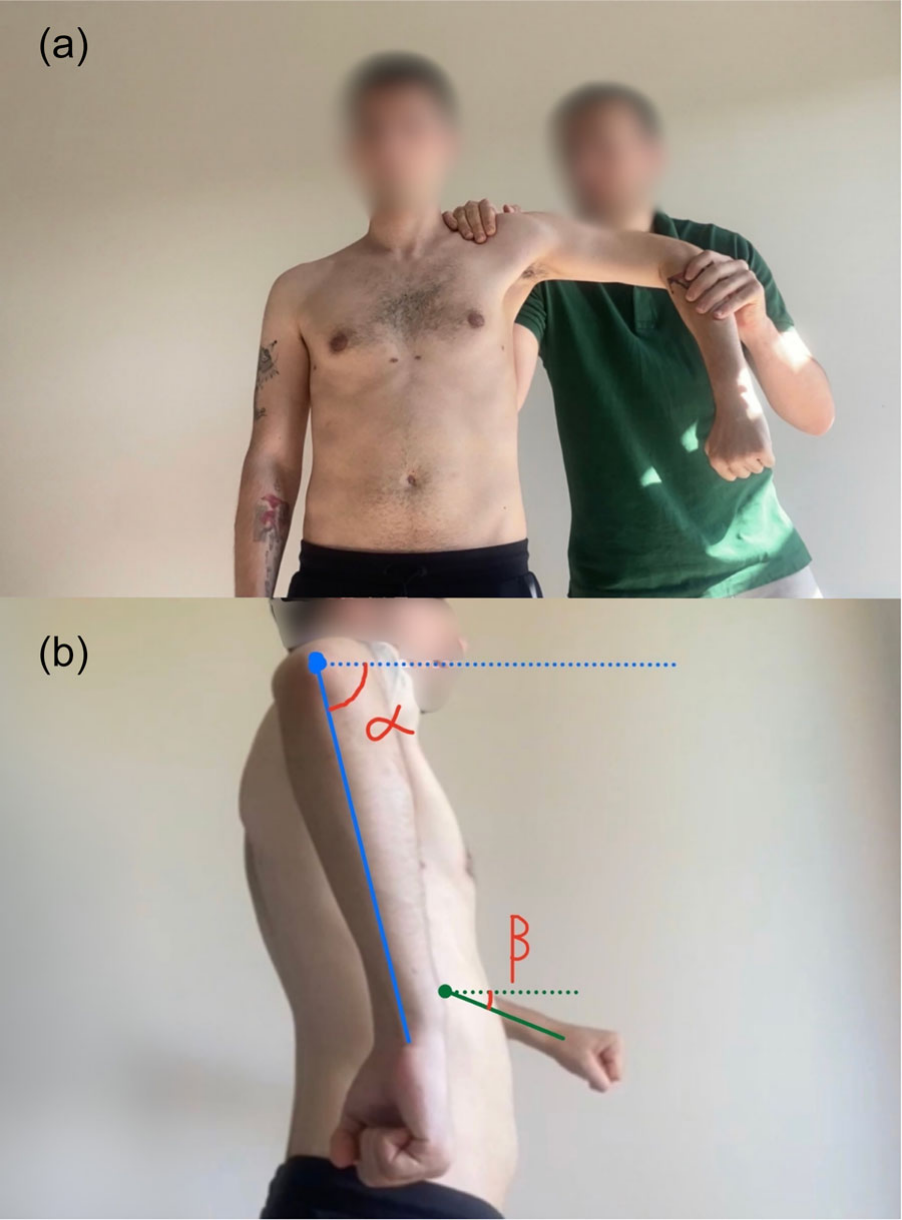
The glenohumeral internal rotation deficit was assessed comparatively (a), revealing a deficit in the left shoulder in this examination (β < α) (b).

**Figure 2:**
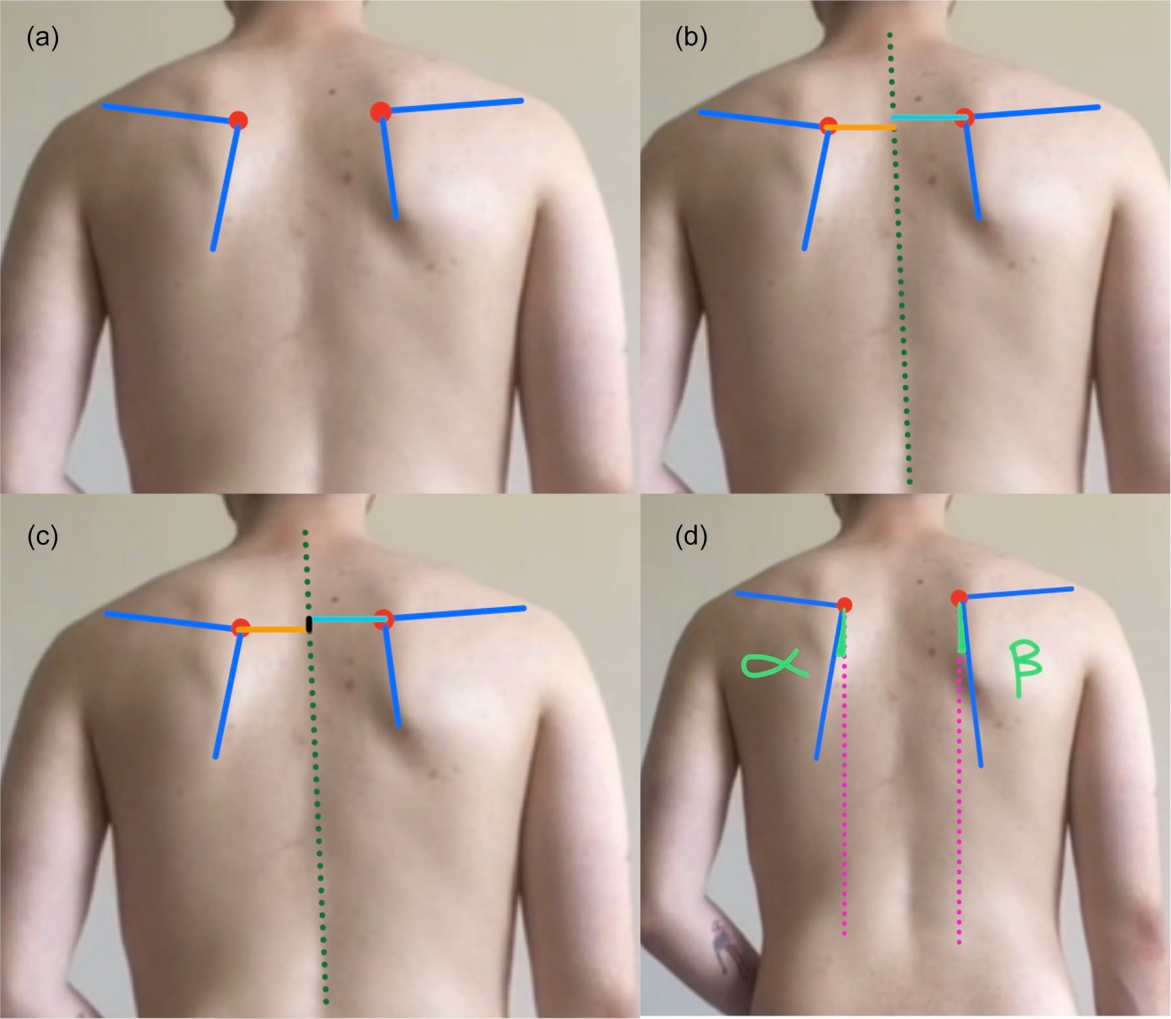
Scapular malposition was assessed as demonstrated. The SICK syndrome (Scapular malposition, Inferior medial border prominence, coracoid pain and malposition and dyskinesis of scapular movement) was evaluated with the SICK Scapula Rating Scale. The blue lines represent the scapular spine and medial scapular border of the scapula, forming the superomedial scapular angle, which is indicated by the red dots (a). The distance of the superomedial scapular angles from the midline (green striped line) is compared between the injured and contralateral scapulae (b). Difference (depicted in black) in vertical height of superomedial scapular angle of injured scapula compared with contralateral superomedial angle (c). Difference in angular degrees of medial scapular border from plumb midline between healthy scapula (α) and injured scapula (β) measured with goniometer (d).

Visual combined palpation was used to classify scapular position and movement patterns (single patterns or mixed patterns) in both the raising and the lowering phases, according to Kibler's method [31]. Visual combined palpation ratings were determined at the time of testing based on classification of the scapular position and movement pattern into four main patterns: [31, 35, 38] Type I, marked by a prominent inferior angle of the scapula; Type II, characterised by prominence along the medial border; Type III, where the upper border of the scapula is prominent; and Type IV, which reflects symmetrical scapular movement. As described by Burkhart et al., the glenohumeral internal rotation deficit was assessed comparatively (Figure 1) and the SICK syndrome (scapular malposition, Inferior medial border prominence, Coracoid pain and malposition and dyskinesis of scapular movement) was evaluated with the SICK scapula rating scale [6] (Figure 2).

**Figure 3:**
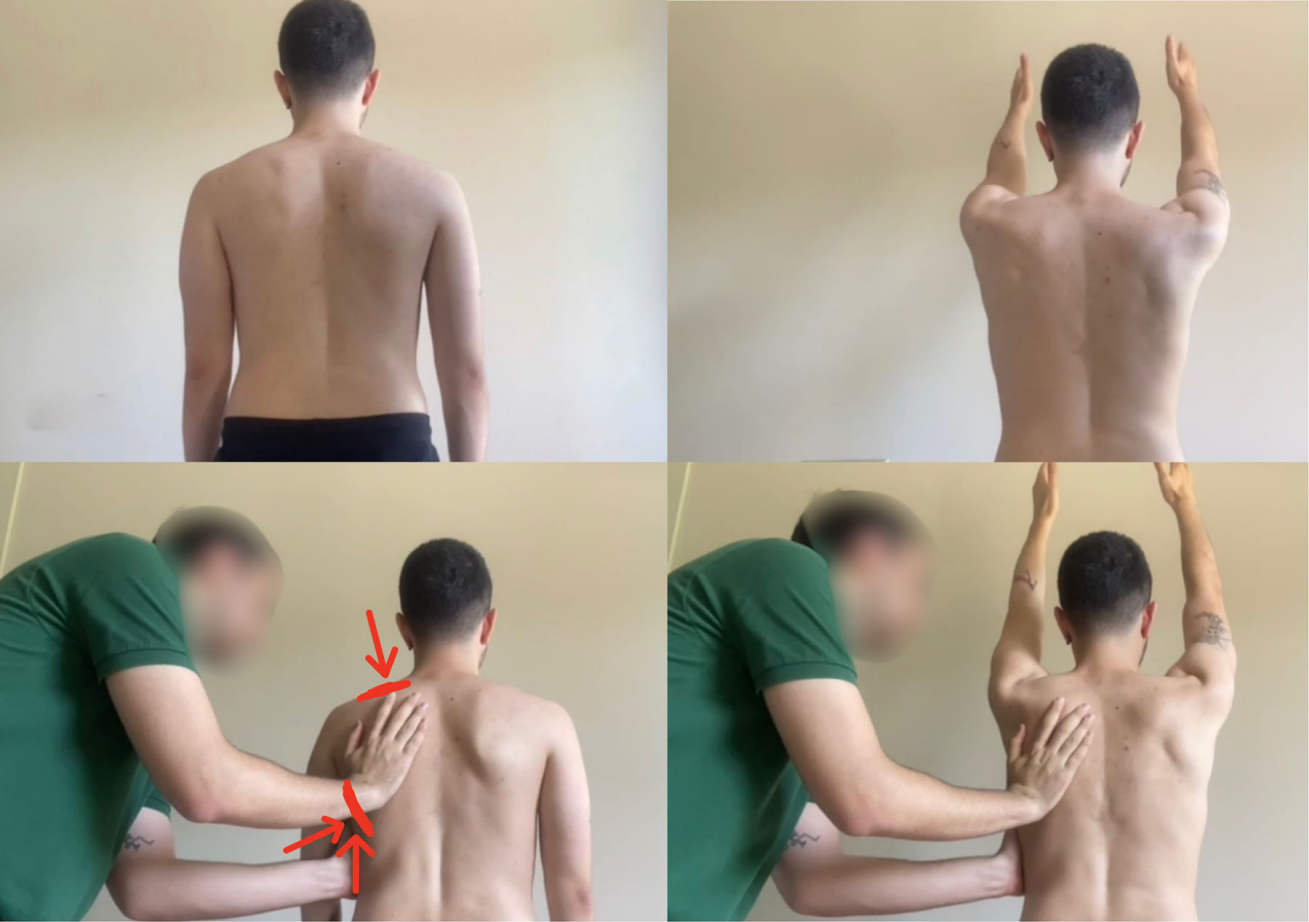
Scapular assistance test (SAT). The examiner guided the scapula manually into upward rotation and posterior tilt, while the participant actively elevated their arm.

Symptom modification tests were used to evaluate the scapular motion [50]. One such test, the scapular assistance test (SAT), involved the examiner manually guiding the scapula into upward rotation and posterior tilt while the participant actively elevated their arm (Figure 3) [25, 53]. The SAT was considered positive if assisted elevation resulted in a reduction of at least two points on the VAS compared to unassisted elevation [53].

**Figure 4:**
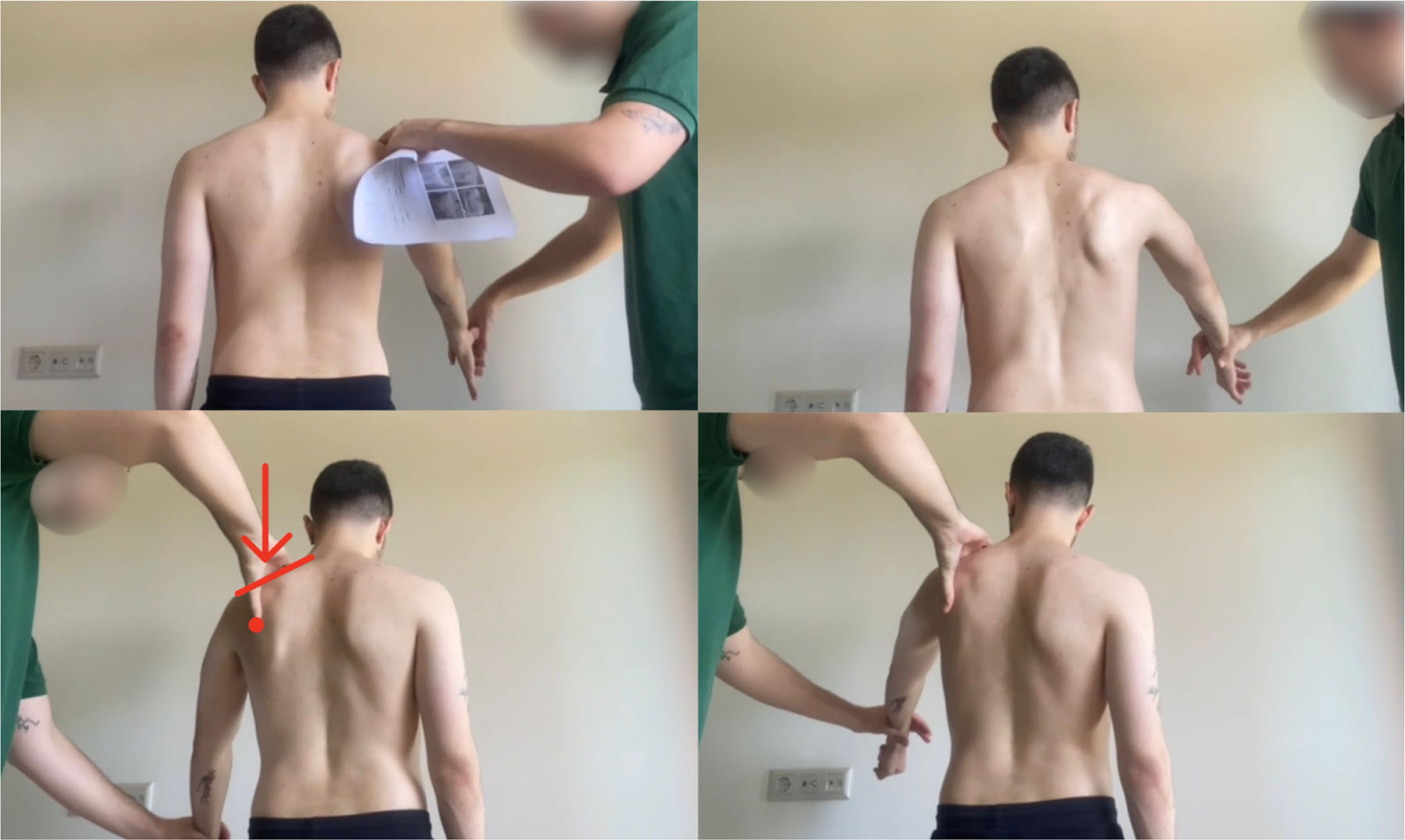
Scapular retraction test (SRT). The examiner stabilised the scapula in a retracted position against the scapula while conducting the standard manual supraspinatus strength assessment using the empty can test, with the arm positioned in the scapular plane.

Kibler et al. introduced the scapular retraction test (SRT) to demonstrate that patients with both rotator cuff dysfunction and SD could experience improved supraspinatus strength deficits when the scapula is stabilised in a retracted position [24, 30]. The test was performed by stabilising the scapula in a retracted position while conducting the manual supraspinatus strength assessment by the examiner using the empty can test, with the arm positioned in the scapular plane (Figure 4). A positive test indicated SD, as evidenced by improved supraspinatus strength compared to the empty can test performed without scapular stabilisation [26, 27].

Finally, the clinical examination was completed by evaluating potential coexisting instability (apprehension test, Kim/Jerk and O'Brien tests) [3, 32, 44] and hyperlaxity (Beighton score) [2].

### Data management and statistical analysis

A descriptive analysis of the variables was performed by calculating mean, median, standard deviation (±), absolute and percentage frequency, for which IBM SPSS Statistics 29.0 software (IBM) was employed. Parametric data were analysed using independent samples *t*‐tests, while nonparametric data were assessed using the Mann–Whitney *U*‐test. Chi‐square tests were applied for dichotomous data. Subgroup analyses were performed on 108 shoulders of 54 participants to assess shoulder function scores and ROM based on the presence of SD, years of experience (cut‐off: 10 years), sex, previous shoulder injury, shoulder pain in the last 12 months, and training intensity (cut‐off: 14 h/week). A *p*‐Value < 0.05 was considered significant.

## RESULTS

All demographics of the participants are illustrated in Table 1. The mean age of all participants (27 female, 27 male) was 23.9 ± 6.5 years. SD was identified in 28.7% of shoulders (31/108; right: *n* = 12, left: *n* = 19), with no participants previously diagnosed with SD. Of the participants with SD, 38.7% had SD on the dominant side (12/31; right: *n* = 9; left: *n* = 3), while the remaining participants had it on the nondominant side. The most common type of SD according to Kibler observed was type II (54.8%; 17/31), followed by type I (41.9%; 13/31) and type III (3.2%; 1/31).

**Table 1:** Patient demographics.

Patient demographics	Mean value	Standard deviation (±)
Sex (*n*)		
Female	27	‐
Male	27	‐
Hand dominance (*n*)		
Right	48	‐
Left	6	‐
Age at examination (years)	23.9	6.5
BMI (kg/m²)	22.8	2.6
Current professional level (*n*)		
EuroLeague	13	‐
EuroCup	29	‐
FIBA National Basketball	12	‐
Playing position (*n*)		
PG	7	‐
SG	3	‐
PG/SG	11	‐
SF	4	‐
SF/PF	10	‐
PF	8	‐
C	11	‐
Experience (years)	11.2	6.0
Training (h/day)	2.4	0.7
Training (h/week)	14.9	3.9

*Note*: Values reported as means with standard deviations (±). Total number of patients: 54.

Abbreviations: BMI, body mass index; C, centre; PF, power forward; PG, point guard; SF, shooting forward; SG, shooting guard.

Nine participants (9/54; 16.7%) gave positive responses to the question if they had over the last 12 months any pain and/or difficulty moving their shoulders that lasted more than a day. Their mean VAS at rest was 2.1 ± 1.5 and mean VAS for exertional pain while performing averaged 4.7 ± 2.0. Of these, seven participants (7/9; 77.8%) had SD.

Shoulders with SD exhibited a significantly lower SSV (95.0 ± 10.5% vs. 99.0 ± 4.0%; *p* = 0.004) and a significantly lower abduction (171.8 ± 11.7° vs. 176.6 ± 8.3°, *p* = 0.013) compared to shoulders without SD (Table 2). There was no significant difference in the prevalence of SD between the shoulders of less and more experienced participants (*p* = 0.136) or between female and male participants (*p* = 0.832). Shoulders with at least one previous injury showed a significantly lower SSV compared to shoulders without previous injury (92.9 ± 12.0% vs. 98.4 ± 5.6%; *p* = 0.001) (Table 3). Shoulders with pain occurring at least once in last 12 months showed a significantly higher prevalence of SD (6/10 vs. 25/98; *p* = 0.022) and a lower SSV (90.5 ± 16.4% vs. 98.6 ± 4.4%; *p* = 0.024) compared to shoulders without pain in last 12 months (Table 4). A higher prevalence of SD was observed in athletes training ≥14 h/week, although the association was not statistically significant (*p* = 0.063) (Table 5).

**Table 2:** Subgroup analysis of clinical outcome between shoulders with and without SD.

Value			
Clinical outcome	With SD (*n* = 31)	Without SD (*n* = 77)	*p*‐Value
SSV (%)	95.0 ± 10.5	99.0 ± 4.0	**0.004**
ABD (°)	171.8 ± 11.7	176.6 ± 8.3	**0.013**
FF (°)	175.5 ± 7.8	177.6 ± 4.1	0.205
IR at 0° ABD (points)	5.8 ± 1.6	5.7 ± 1.5	0.901
ER at 0° ABD (°)	68.8 ± 16.5	69.0 ± 15.0	0.970
IR at 90° ABD (°)	66.8 ± 18.3	68.3 ± 16.5	0.488
ER at 90° ABD (°)	95.1 ± 10.5	93.4 ± 11.5	0.623
Beighton score (points)	2.4 ± 2.0	2.2 ± 2.1	0.659

*Note*: Values as means reported with standard deviations (±). Statistically significant *p*‐Values are highlighted in bold.

Abbreviations: ABD, abduction; ER, external rotation; FF, forward flexion; IR, internal rotation; SD, scapular dyskinesis; SSV, subjective shoulder value.

**Table 3:** Subgroup analysis of clinical outcome between shoulders with and without previous injury.

Value			
Clinical outcome	No previous shoulder injury (*n* = 96)	At least one previous shoulder injury present (*n* = 12)	*p*‐Value
Existing SD (*n*)	25	6	0.084
SSV (%)	98.4 ± 5.6	92.9 ± 12.0	**0.001**
ABD (°)	175.0 ± 9.9	177.1 ± 6.9	0.477
FF (°)	176.9 ± 5.7	177.9 ± 3.5	0.726
IR at 0° ABD (points)	5.7 ± 1.5	6.3 ± 1.8	0.233
ER at 0° ABD (°)	69.1 ± 14.8	67.9 ± 20.3	0.918
IR at 90° ABD (°)	67.5 ± 16.8	71.2 ± 18.6	0.277
ER at 90° ABD (°)	93.4 ± 10.9	93.4 ± 14.1	0.825
Beighton score (points)	2.2 ± 2.0	2.9 ± 2.1	0.177

*Note*: Values as means reported with standard deviations (±). Statistically significant *p*‐Values are highlighted in bold.

Abbreviations: ABD, abduction; ER, external rotation; FF, forward flexion; IR, internal rotation; SD, scapular dyskinesis; SSV, subjective shoulder value.

**Table 4:** Subgroup analysis of clinical outcome between shoulders with and without previous pain in last 12 months.

Value			
Clinical outcome	No shoulder pain in last 12 months (*n* = 98)	Shoulder pain in last 12 months present (*n* = 10)	*p*‐Value
Existing SD (*n*)	25	6	**0.022**
SSV (%)	98.6 ± 4.4	90.5 ± 16.4	**0.024**
ABD (°)	175.4 ± 9.7	173.5 ± 8.5	0.322
FF (°)	177.1 ± 5.5	176.6 ± 5.3	0.760
IR at 0° ABD (points)	5.8 ± 1.5	5.4 ± 1.0	0.592
ER at 0° ABD (°)	69.4 ± 14.6	64.3 ± 21.8	0.576
IR at 90° ABD (°)	68.3 ± 16.6	63.8 ± 20.6	0.584
ER at 90° ABD (°)	94.1 ± 10.4	91.5 ± 17.7	0.705
Beighton score (points)	2.2 ± 2.1	2.7 ± 1.6	0.275

*Note*: Values as means reported with standard deviations (±). Statistically significant *p*‐Values are highlighted in bold.

Abbreviations: ABD, abduction; ER, external rotation; FF, forward flexion; IR, internal rotation; SD, scapular dyskinesis; SSV, subjective shoulder value.

**Table 5:** Subgroup analysis of clinical outcome between shoulders with less and more training intensity.

Value			
Clinical outcome	Less training (*n* = 50; ≤14 h/week)	More training (*n* = 58; >14 h/week)	*p* Value
Existing SD (n)	10	21	0.063
Existing previous injury (*n*)	3	9	0.117
Existing previous pain in last 12 months (*n*)	4	6	0.675
SSV (%)	97.5 ± 8.9	98.1 ± 4.1	0.125
ABD (°)	176.3 ± 7.3	174.2 ± 11.2	0.517
FF (°)	177.6 ± 3.9	176.5 ± 6.5	0.867
IR at 0° ABD (points)	5.3 ± 1.3	6.1 ± 1.6	**0.003**
ER at 0° ABD (°)	68.3 ± 14.6	69.5 ± 16.1	0.528
IR at 90° ABD (°)	65.5 ± 19.4	70.0 ± 14.5	0.314
ER at 90° ABD (°)	92.5 ± 8.3	95.1 ± 13.2	0.239
Beighton score (points)	1.8 ± 1.7	2.7 ± 2.2	**0.046**

*Note*: Values as means reported with standard deviations (±). Statistically significant *p*‐Values are highlighted in bold.

Abbreviations: ABD, abduction; ER, external rotation; FF, forward flexion; IR, internal rotation; SD, scapular dyskinesis; SSV, subjective shoulder value.

## DISCUSSION

The most important aspect of the study is that the present study identified and assessed the prevalence of SD in overhead professional athletes. SD was observed in 28.7% of the shoulders in asymptomatic European professional basketball players. Moreover, SD may precede the onset of symptoms—particularly in overhead athletes—making its identification in asymptomatic individuals a potential early marker of altered shoulder mechanics. This could help guide preventive strategies before pain or dysfunction develops. In the present study, shoulders with SD exhibited a significantly lower SSV and abduction, although all the participants were asymptomatic at the time of the examination.

In comparison to non‐overhead athletes, overhead athletes demonstrate a significantly greater reported prevalence of SD due to increased forces and stress on the shoulder [7, 58, 59]. Previous studies [7, 45] have demonstrated that athletes in volleyball [59], baseball [40, 49], swimming [37, 60] and handball [9] have an almost double risk of developing SD compared to those in non‐overhead sports. However, not all observed SD is associated with shoulder symptoms and dysfunction [29]. In the present study, 28.7% of the included shoulders of professional basketball players exhibited SD. However, none of the participants had a prior diagnosis of SD before the examination, and only 17% reported shoulder pain and/or difficulty moving their shoulders lasting more than a day in the last 12 months. The presented study evaluated athletes at rest, before any physical effort and at the end of the season, to obtain a realistic prevalence of SD excluding the influence of fatigue.

In the current study, shoulders with SD showed significantly reduced SSV and abduction, despite all participants being asymptomatic at the time of assessment. However, the significant difference in SSV was below the minimal clinically important difference (MCID) described in the literature for arthroscopic massive rotator cuff repair (13.7%) [21] and proximal humerus fractures (12.1%) [12, 62]. Prior studies have demonstrated that altered scapular kinematics associated with pain, such as reduced upward rotation at 45° and 90° of shoulder abduction, can impair shoulder function [10, 15, 58, 59], thus increasing the theoretical risk of injury. Hickey et al. demonstrated in their systematic review and meta‐analysis that asymptomatic athletes with SD have an increased risk of future shoulder pain by 43% [16]. Early recognition of SD enables therapeutic interventions, such as scapular‐focused exercises, which have been shown to reduce symptoms, improve shoulder function and mitigate injury risks [15, 58].

Shoulders with at least one previous injury, and pain occurring at least once in the last 12 months exhibited a significantly lower SSV. However, there were no other statistically significant relevant differences in the subgroup analyses, making it impossible to correlate the examined factors with SD. These findings support the idea that SD may reflect a sport‐specific adaptation in overhead athletes [11, 36]. Alternatively, SD may serve as a risk factor that identifies athletes at a higher risk of injury. Two prospective studies investigated whether SD in asymptomatic athletes increases the risk of developing shoulder pain, but the results were inconclusive [9, 39]. Therefore, more studies with larger sample size are needed to determine whether the presence of SD predisposes one to developing SD with or without shoulder symptoms or whether SD is just a functional adaptation that helps the athlete have a more efficient performance.

Although the group with higher training intensity had more cases of SD and preexisting shoulder injuries, training intensity itself was not significantly associated with SD (*p* = 0.063). High training volume may contribute to shoulder injuries, which could, in turn, lead to a higher prevalence of SD in athletes [57]. Additionally, SD may result from muscular fatigue due to excessive training loads. Madsen et al. evaluated 78 asymptomatic swimmers and demonstrated that the prevalence of SD during a normal training session is high; it increased with more training and occurred early during the training session [37]. However, Crotty and Smith observed no significant scapular alterations pre‐ and postexercise in male high school swimmers undergoing intense training [43]. Further research with larger sample sizes and matched‐pair analyses is needed to determine whether SD correlates with training intensity.

Different sport‐specific gestures could be associated with a higher risk of developing a sport specific SD pattern. Using the descriptive classification of SD described by Kibler et al. [31], the present study showed that type II is the most frequent pattern in professional basketball players with SD (54.8%), followed by type I (41.9%) and type III (3.2%). No previous studies have investigated the prevalence of SD patterns among professional basketball players. Standoli et al. demonstrated among 661 asymptomatic elite swimmers I, II and III were present in 46.5%, 35.7% and 17.8% of cases, respectively [52]. Kawasaki et al., investigating a cohort of 103 elite rugby players, found that types I, II and III were present in 4.9%, 3.9% and 22.2% of cases, respectively [23].

The strong part of the study is that this is the first prospectively recruited cross‐sectional study on SD which was performed in a large group of European basketball players. While SD may represent an adaptation to overhead sports, our findings show a higher proportion of SD in athletes reporting previous injuries and shoulder pain in the last 12 months. However, no direct correlation was found with training intensity. Routine screening for SD is recommended, as early recognition and scapular‐focused exercises could improve shoulder function and help prevent future injuries.

The current study has some limitations. First, the lack of three‐dimensional motion analysis, which is considered the gold standard for kinematic assessment, limits the precision of SD evaluation. Although manual goniometry is a well‐established method for assessing ROM in the upper extremities when performed in a standardised manner, 3D motion capture remains the gold standard according to the recommendations of the International Society of Biomechanics [17]—a method that is lacking in the present study. Second, the literature describes considerable heterogeneity in the assessment of SD [18, 34, 48]; therefore, involving more than one examiner and conducting evaluations at two independent time points would have improved the validity of the assessment in this study [33]. Third, the reliance on self‐reported shoulder pain and injuries over the past 12 months can be seen as a limitation, which may be subject to recall bias and affect the accuracy of symptom history. Fourth, due to the limited availability of professional athletes—given their training schedules and other commitments—all eligible players available during the recruitment period were included, and a formal power analysis was not performed in advance. The aim was to maximise the sample size within these practical constraints. Fifth, unbalanced subgroup distribution limits the generalisability of findings to all professional basketball players. Further research with sufficient sample size for subgroup analysis or with a homogeneous type of SD is needed to observe athletes longitudinally over time to see if participants with SD develop shoulder symptoms and to set up a rehabilitation protocol.

## CONCLUSION

SD was present in 28.7% of shoulders among asymptomatic European professional basketball players. While SD may represent a sport‐specific adaptation, its association with reduced SSV, diminished abduction and reported shoulder pain over the last 12 months underscores its clinical significance. Routine screening for SD during health examinations may facilitate timely interventions, such as scapular‐focused exercises, to enhance shoulder function and mitigate the risk of future injuries.

## AUTHOR CONTRIBUTIONS

**Alp Paksoy**: Conceptualisation; methodology; software; validation; formal analysis; original draft preparation; review and editing; visualisation. **Doruk Akgün**: Conceptualisation; methodology; validation; original draft preparation; supervision; project administration. **Jonas Pawelke**: Conceptualisation; methodology. **Larissa Eckl**: Conceptualisation; methodology. **Arda Mavi**: Software; visualisation. **Selda Uzun**: Investigation; resources; data curation. **Berhan Bayram**: Investigation; resources; data curation. **Murat Canbakal**: Investigation; resources; data curation. **Ugur Dilicikik**: Investigation; resources; data curation. **Murat Erdem**: Investigation; resources; data curation. **Nihat D. Demirkiran**: Investigation; resources; data curation; review and editing; supervision. **Baris Kocaoglu**: Conceptualisation; methodology; validation; formal analysis; investigation; resources; data curation; original draft preparation; review and editing; supervision; project administration. All authors have read and agreed to the published version of the manuscript.

## CONFLICT OF INTEREST STATEMENT

The authors declare no conflicts of interest.

## ETHICS STATEMENT

Ethical approval from the institutional ethics committee of Charité Universitätsmedizin was obtained prior to onset of investigation (Application number: EA4/024/25; Date of approval: 08.04.2025). Informed consent was obtained from all subjects involved in the study.

## Data Availability

The data that support the findings of this study are available from the corresponding author upon reasonable request.
